# Is soy intake related to age at onset of menarche? A cross-sectional study among adolescents with a wide range of soy food consumption

**DOI:** 10.1186/1475-2891-13-54

**Published:** 2014-06-03

**Authors:** Gina Segovia-Siapco, Peter Pribis, Mark Messina, Keiji Oda, Joan Sabaté

**Affiliations:** 1 Department of Nutrition, Loma Linda University, 24951 North Circle Dr., Nichol Hall 1105, Loma Linda, CA 92350, USA; 2 Department of Biostatistics and Epidemiology, School of Public Health, Loma Linda University, 24591 No. Circle Drive, Loma Linda, CA 92350, USA; 3 School of Health Professions, Department of Public Health & Wellness, Andrews University, 8475 University Boulevard – Marsh Hall, Berrien Springs, MI 49104, USA; 4 Nutrition Matters, Inc, 439 Calhoun Street, Port Townsend, WA 98368, USA

**Keywords:** Soy, Adolescence, Age at menarche, Pubertal timing, Puberty, Seventh-day Adventists

## Abstract

**Background:**

Early onset of menarche may negatively influence the future health of adolescent girls. Several factors affect the timing of menarche but it is not clear if soy foods consumption around pubertal years plays a role; thus, we examined its relation to age at onset of menarche (AOM) in a high soy-consuming population.

**Methods:**

We conducted a cross-sectional study on 339 girls ages 12–18 years attending middle and high schools near two Seventh-day Adventist universities in California and Michigan using a web-based dietary questionnaire and physical development tool. Soy consumption (categorized as *total soy, meat alternatives, tofu/traditional soy,* and *soy beverages*) was estimated from the questionnaire, while AOM was self-reported. Data analyses included descriptive statistics, Cox proportional hazards ratios, Kaplan-Meier curves and Poisson regression with adjustment for relevant confounders.

**Results:**

Mean (SD) intakes were: total soy,12.9 (14.4) servings/week; meat alternatives, 7.0 (8.9) servings/week; tofu/traditional soy foods, 2.1 (3.8) servings/week; soy beverages, 3.8 (6.3) servings/week. Mean AOM was 12.5 (1.4) y for those who reached menarche. Consumption of total soy and the 3 types of soy foods was not significantly associated with AOM and with the odds for early- or late-AOM. Adjustment for demographic and dietary factors did not change the results.

**Conclusion:**

Soy intake is not associated with AOM in a population of adolescent girls who have a wide range of, and relatively higher, soy intake than the general US population. Our finding suggests that the increasing popularity of soy in the US may not be associated with AOM.

## Background

For the past four decades, age at which puberty occurs among girls—manifested as breast development, appearance of pubic hair, and onset of menarche—has been commencing earlier. Although evidence points to non-linear variability in menarcheal age from decade to decade [1], the mean age at onset of menarche (AOM) in the United States decreased by an average of 3 to 5½ months from 1960 to 1990 [2]. Breast development, typically considered an earlier manifestation of puberty compared to menarche, is also commencing at a younger age [3]. The secular trends in pubertal development raise concerns because early onset of puberty may negatively influence the future health of adolescent girls [4]. Earlier menarche has been implicated in the etiology of breast and ovarian cancer [5,6]; in fact, early menarche has been found to contribute more to breast cancer risk than late menopause [7]. Therefore, the decline in age at which menarche occurs may result in an increased incidence of hormone-related cancers.

Several investigations have focused on the role of genetics [8,9], early life exposure to environmental factors and xenobiotics [10-12], and body composition/weight status [13-16] on pubertal development. Certain dietary factors, such as animal protein and meat [17,18] and milk and total dairy [19], have also been linked to pubertal timing. Over the past two decades, foods made from soybeans have increased in popularity in many western countries due in part to awareness of the proposed health benefits of these foods. Soy foods are sources of high-quality protein, substantial amounts of essential fatty acids, isoflavones, and in some cases, dietary fiber. Isoflavones bind to estrogen receptors and exert estrogen-like effects under certain conditions [20], thus, isoflavone exposure may affect sexual maturation of children [21]. It is, therefore, not surprising that some studies investigating pubertal development have focused on soy isoflavones.

The few investigations on the effects of exposure to soy early in life on the onset of menarche have produced mixed results [22-24]. Given the possible hormonal effects of soy and the decline in age at onset of puberty in the US – although this decline has also occurred in countries not exposed to soy – there is clearly a need for additional soy and isoflavone research in this area. However, isoflavones are only one of the many components of soy that may also be associated with pubertal timing. Evidence suggests that vegetable and animal protein may have differential effects on pubertal development [25-27]. In addition, dietary fiber and fat have been found to influence the menarcheal age [28-30]. Thus, the possible synergism among soy components and their cumulative effect on pubertal development needs to be considered.

The Adventist population offers an opportunity to study the relationships between intake of soy foods and AOM because a high percentage of them are vegetarians and include soy-containing foods in their diet. In fact, our preliminary data (unpublished) from a pilot study among adolescents attending Adventist schools or public schools near major Adventist universities in Southern California and Michigan, revealed a wide range in the consumption of soy-containing foods. Therefore, our aim was to determine if soy intake is associated with the AOM in a population that is known to have extensive exposure to soy.

## Methods

### Study design and participants

The Teen Food and Development Study is a cross-sectional study conducted using a web-based survey to determine the relationships between food intake and pubertal development. In particular, we sought to determine how soy consumption is related to the AOM. The study participants were recruited from Adventist and public middle and high schools near major Adventist universities in southern California and Michigan. Parents and prospective participants enrolled online through a website that was designed specifically for the study. Parental permission was granted by filling out the online informed consent form together with the child who also filled out the form. To ascertain that the parent indeed approved the child’s participation, a confirmation call to the parent was made. The online mode of collecting informed consent by the parents, assent of the potential participant, and all study protocols were approved by the Institutional Review Boards of Loma Linda University and Andrews University.

Assuming a unidirectional relationship between soy consumption and AOM, we determined *a priori* that a sample size of 340 will yield 75% power to detect a 3-month difference in AOM between consumers and non-consumers of soy. Adolescent girls 12 to 18 years old were included in the study. We limited the study population to this age range because of the likelihood that they have already reached menarche and to cover a wider range of ages at which menarche might occur. Menarche is considered as a late manifestation of puberty compared to thelarche and pubarche [31], but since menarche is regarded as an important event in the life of a teenage girl, this pubertal marker is most accurately remembered [32]. A total of 339 girls completed the survey. Twelve were not included in the analysis: 9 have missing data on AOM and 3 had very high and/or improbable values for intake of soy foods and total food intake. Thus, 327 adolescent girls with complete information on soy intake and AOM were considered eligible for this study.

### Assessment and validation of soy consumption

The self-administered web-based survey—which was pre-tested on a representative group (n = 18) of adolescent girls of the same age for clarity, comprehension, and relevance—included a semi-quantitative food frequency questionnaire (FFQ) composed of 151 food items (see Additional file 1). Food items were categorized as *convenience foods* (32 items), *protein-rich foods* (29 items), *starches/cereals* (17 items), *vegetables/fruits* (21 items), *dairy products* (10 items), *beverages* (24 items), *snacks/sweets* (11 items), and *soups/legumes* (7 items). Participants were asked to self-report frequency of their intake during the previous 6 months. Frequency of intake categories were: never/rarely, 1–3 times per month, once per week, 2–4 times per week, 5–6 times per week, once per day, 2–3 times per day, and ≥4 times per day. The FFQ had 36 soy-containing foods, most of which are meat or dairy substitutes. Soy foods were grouped into three categories: *meat alternatives, tofu/traditional soy,* and *soy beverages/dairy substitutes*.

Soy consumption using the web-based FFQ was validated relative to 6 food records which were provided by each of a subgroup of participants (n = 70) from both study sites. Corrected correlation coefficient for intake of soy-containing foods was 0.63 (95% bootstrap BCa CI: 0.43, 0.77). Total agreement (+/- 1 quartile) between the two methods was 83% while gross misclassification was 2.9%. Thus, we considered the web-based FFQ to provide a valid estimate of soy foods consumption and in ranking individuals according to their intake.

### Assessment of AOM

The web-based survey included questions on physical and pubertal development. The AOM was self-reported by answering the question, “What was your age when you had your first-ever period?” where participants were asked to report their age in years to the nearest number of months (e.g., 11 years and 9 months). At the time of the survey, 293 participants reported that they were already menstruating while 34 (~10%) had not yet menstruated.

### Data management and analysis

Descriptive analysis was performed for the demographic profile of the participants. Tests to compare distributions and means or medians (i.e., χ^2^, *t*-test, one-way ANOVA, and Kruskal-Wallis test) among the soy intake categories were performed testing at α = 0.05. Since some of the participants had not yet reached menarche (~10%), we did time-to-event analyses to determine differences in AOM among the different consumption levels of *total soy, meat alternatives, tofu/ traditional soy,* and *soy beverages/dairy substitutes*. Cox proportional hazards regression/modeling was performed to estimate the hazard of reaching menarche at any given age using both continuous and categorical levels of soy exposure. Kaplan-Meier curves were plotted to check if there were observable differences in the timing of AOM between low- (below median of total soy consumption) and high- (above median of total soy consumption) soy consumers using crude energy-adjusted total soy intake. In addition, we conducted modified Poisson regression modeling to estimate the relative risk for early and late menarche among the levels of soy consumption groups. Early-onset menarche was defined as AOM < 12.0 years and late-onset menarche as AOM ≥ 14.0 years which roughly coincided with the 25^th^ and 75^th^ percentiles, respectively, of the AOM distribution of the study participants. The 25^th^ and 75^th^ percentiles have been used as cut-points in other studies to define the timing of menarche [33].

Soy consumption was measured as frequency and as servings of soy foods and total food intake was expressed as the number of servings of all foods consumed per day. A crude adjustment for energy intake was done by dividing the number of servings of soy by the number of servings of total food intake. From the adjusted values, quartiles of *total soy* and *meat alternatives* consumption were compared using the lowest quartile group (Quartile 1) as the reference. Due to more skewed energy-adjusted intake distributions of tofu/traditional soy and soy beverages/dairy substitutes, levels of intake were divided into non-consumers (“0” soy consumption), low-to-moderate consumers (≤75th percentile but excluding non-consumers) and high consumers (>75^th^ percentile); non-consumers was used as the reference.

Models were adjusted for covariates which were significantly distributed differently among the levels of soy consumption groups – age, study site, type of school, socio-economic status (measured in terms of mother’s and father’s educational levels), ethnicity (measured in terms of mother’s and father’s ethnicities) – and those purported to be associated with age of menarche, such as meat and total food intake, or mediate the association between food intake and AOM, such as body mass index z-score (BMIz). An interaction term between soy intake and BMIz was initially included in all models and found not significant, thus, was excluded from the final model. All analyses were performed using SAS 9.3 (SAS Institute Inc., Cary, NC, USA) at an alpha level of 0.05.

## Results

Table 1 presents the participants’ characteristics according to their soy intake categories. Age distributions and mean ages were similar across soy consumption levels. Of the 327 participants, 46.5 and 53.5% were from Michigan and California, respectively. Most study participants have highly educated parents, the majority of whom (about 80%) had at least a four-year college degree. More than 40% of the parents were Caucasian while Hispanics, Blacks, Asians, and other ethnicities accounted for 18-20%, 10-12%, 14-15%, and 11%, respectively. There were statistically significant distribution differences across the levels of soy consumption in terms of site, type of school, mother’s and father’s education, and mother’s and father’s ethnicities. Approximately 24% of the participants never or rarely eat meat, and meat intake significantly differed across soy intake groups (p < 0.0001). In contrast, BMI and BMIz did not differ significantly across the soy consumption categories. Distribution of AOM and the unadjusted mean AOM of those who have reached menarche (overall, 12.5 ± 1.4 y) did not significantly differ across levels of soy consumption. Similar non-significant differences were also found for *meat alternatives, tofu/traditional soy,* and *soy beverages/dairy substitutes* (not shown).

**Table 1:** Demographic characteristics of participants as a whole and according to categories of total soy consumption level

	**Total (n = 327) count (%)**	**Level of soy consumption (frequency of intake)**				**p-value**
		**≤1×/week (n = 62) count (%)**	**>1×/week - ≤1×/day (n = 85) count (%)**	**>1×/day - ≤3×/day (n = 111) count (%)**	**>3×/day (n = 69) count (%)**	
**Age, years**						0.574^b^
12	26 (8.0%)	6 (9.7%)	7 (8.2%)	9 (8.1%)	4 (5.8%)	
13	51 (15.6%)	8 (12.9%)	13 (15.3%)	19 (17.1%)	11 (15.9%)	
14	56 (17.1%)	9 (14.5%)	17 (20.0%)	19 (17.1%)	11 (15.9%)	
15	61 (18.7%)	7 (11.3%)	18 (21.2%)	19 (17.1%)	17 (24.6%)	
16	59 (18.0%)	14 (22.6%)	8 (9.4%)	21 (18.9%)	16 (23.2%)	
17	50 (15.3%)	14 (22.6%)	15 (17.6%)	14 (12.6%)	7 (10.1%)	
18	24 (7.3%)	4 (6.5%)	7 (8.2%)	10 (9.0%)	3 (4.3%)	
**Mean age (SD), years**	15.0 (1.5)	15.2 (1.8)	14.9 (1.8)	14.9 (1.8)1	14.9 (1.5)	0.771^c^
**Site**						0.006^b^
Michigan	152 (46.5%)	41 (66.1%)	36 (42.4%)	49 (44.1%)	26 (37.7%)	
California	175 (53.5%)	21 (33.9%)	49 (57.6%)	62 (55.9%)	43 (62.3%)	
**School**						<.0001^b^
Public	38 (11.6%)	23 (37.1%)	8 (9.4%)	4 (3.6%)	3 (4.3%)	
Private	289 (88.4%)	39 (62.9%)	77 (90.6%)	107 (96.4%)	66 (95.7%)	
**Mother’s education**						0.0008^b^
High school level/graduate	63 (19.3%)	24 (38.7%)	13 (15.3%)	13 (11.7%)	13 (18.8%)	
College level/degree	133 (40.7%)	26 (41.9%)	41 (48.2%)	50 (45.0%)	30 (43.5%)	
Masters/Doctoral level/degree	131 (40.1%)	12 (19.4%)	31 (36.5%)	48 (43.2%)	26 (37.7%)	
**Father’s education**						0.0001^b^
High school level/graduate	68 (20.8%)	24 (38.7%)	21 (24.7%)	13 (11.7%)	10 (14.5%)	
College level/degree	113 (34.6%)	28 (45.2%)	35 (41.2%)	43 (38.7%)	21 (30.4%)	
Masters/Doctoral level/degree	146 (44.6%)	10 (16.1%)	29 (34.1%)	55 (49.5%)	38 (55.1%)	
**Mother’s ethnicity**						0.0004^b^
White	142 (43.4%)	35 (56.5%)	34 (40.0%)	52 (46.8%)	21 (30.4%)	
Black	34 (10.4%)	3 (4.8%)	10 (11.8%)	13 (11.7%)	8 (11.6%)	
Hispanic	65 (19.9%)	15 (24.2%)	15 (17.6%)	16 (14.4%)	19 (27.5%)	
Asian	50 (15.3%)	3 (4.8%)	8 (9.4%)	22 (19.8%)	17 (24.6%)	
Other	36 (11.0%)	6 (9.7%)	18 (21.2%)	8 (7.2%)	4 (5.8%)	
**Father’s ethnicity**						0.004^b^
White	144 (44.0%)	36 (58.1%)	31 (36.5%)	53 (47.7%)	24 (34.8%)	
Black	41 (12.5%)	4 (6.5%)	14 (16.5%)	15 (13.5%)	8 (11.6%)	
Hispanic	60 (18.3%)	12 (19.4%)	15 (17.6%)	16 (14.4%)	17 (24.6%)	
Asian	46 (14.1%)	2 (3.2%)	9 (10.6%)	20 (18.0%)	15 (21.7%)	
Other	36 (11.0%)	8 (12.9%)	16 (18.8%)	7 (6.3%)	5 (7.2%)	
**Meat intake, frequency/week**						<.0001^b^
Never	79 (24.2%)	0 (0.0%)	8 (9.4%)	40 (36.0%)	31 (44.9%)	
≤1x/day	111 (33.9%)	17 (27.4%)	38 (44.7%)	39 (35.1%)	17 (24.6%)	
>1x/day	137 (41.9%)	45 (72.6%)	39 (45.9%)	32 (28.8%)	21 (30.4%)	
**Median meat intake (IQR), svg/d**	5.5 (12.5)	13.3 (12.5)	7.0 (8.0)	2.0 (8.0)	1.5 (10.0)	<.0001^d^
**Median BMI (IQR), kg/m**^**2**^	21.9 (4.2)	22.3 (3.7)	22.7 (4.7)	21.3 (4.1)	21.5 (3.8)	0.088^d^
**Mean BMI z-score (SD)**	0.43 (1.02)	0.57 (0.92)	0.62 (1.14)	0.28 (0.98)	0.34 (1.01)	0.084^c^
**Age at onset of menarche (AOM)**						0.498^b^
≤9	13 (4.0%)	3 (4.8%)	4 (4.7%)	4 (3.6%)	2 (2.9%)	
10	27 (8.3%)	6 (9.7%)	10 (11.8%)	7 (6.3%)	4 (5.8%)	
11	39 (11.9%)	2 (3.2%)	12 (14.1%)	13 (11.7% )	12 (17.4%)	
12	122 (37.3%)	30 (48.4%)	26 (30.6%)	43 (38.7%)	23 (33.3%)	
13	48 (14.7%)	7 (11.3%)	11 (12.9%)	19 (17.1%)	11 (15.9%)	
14	33 (10.1%)	5 (8.1%)	8 (9.4%)	10 (9.0%)	10 (14.5%)	
≥15	11 (3.4%)	2 (3.2%)	4 (4.7%)	4 (3.6%)	1 (1.4%)	
Not yet	34 (10.4%)	7 (11.3%)	10 (11.8%)	11 (9.9%)	6 (8.7%)	
**Mean AOM (SD)**^**a**^	12.5 (1.4)	12.5 (1.4)	12.4 (1.6)	12.6 (1.3)	12.6 (1.3)	0.835

^a^Excludes those who have no menarche as yet.

^b^Chi-square test.

^c^One-way ANOVA.

^d^Kruskal-Wallis test.

More than half of the participants (55%) eat soy foods more than once a day (Table 2). Fifty-four percent eat meat alternatives more than 4 times per week. While 23% eat tofu and other traditional soy foods 3 or more times per week, 45% never or rarely eat them. Similarly, 22% drink soy beverages once or more per day while 39% never or rarely do. Meat alternatives comprise most of the weekly soy foods consumed by the participants (mean [SD] = 7.0 [8.9] svg/wk) while soy beverages and tofu/traditional soy foods are eaten in lesser quantities, 3.8 (6.3) vs. 2.1 (3.8) svg/wk, respectively.

**Table 2:** Frequency of intake and mean (SD) consumption of soy foods

**Frequency of intake**	**n**	**%**	**No. servings/week mean (SD)**
**Total soy**			12.9 (14.4)
Up to once/week	62	19.0%	
2-6 times/week	85	26.0%	
1-3 times/day	111	33.9%	
4+ times/day	69	21.1%	
**Meat alternatives**			7.0 (8.9)
Less than once/week	81	24.8%	
1-3 times/week	70	21.4%	
4+ times/week	176	53.8%	
**Tofu/Traditional soy**			2.1 (3.8)
Never/rarely	146	44.6%	
Up to 2 times/week	107	32.7%	
3+ times/week	74	22.6%	
**Soy beverages**			3.8 (6.3)
Never/rarely	128	39.1%	
Up to once/week	56	17.1%	
2-6 times/week	72	22.0%	
1+/day	71	21.7%	

**Figure 1:**
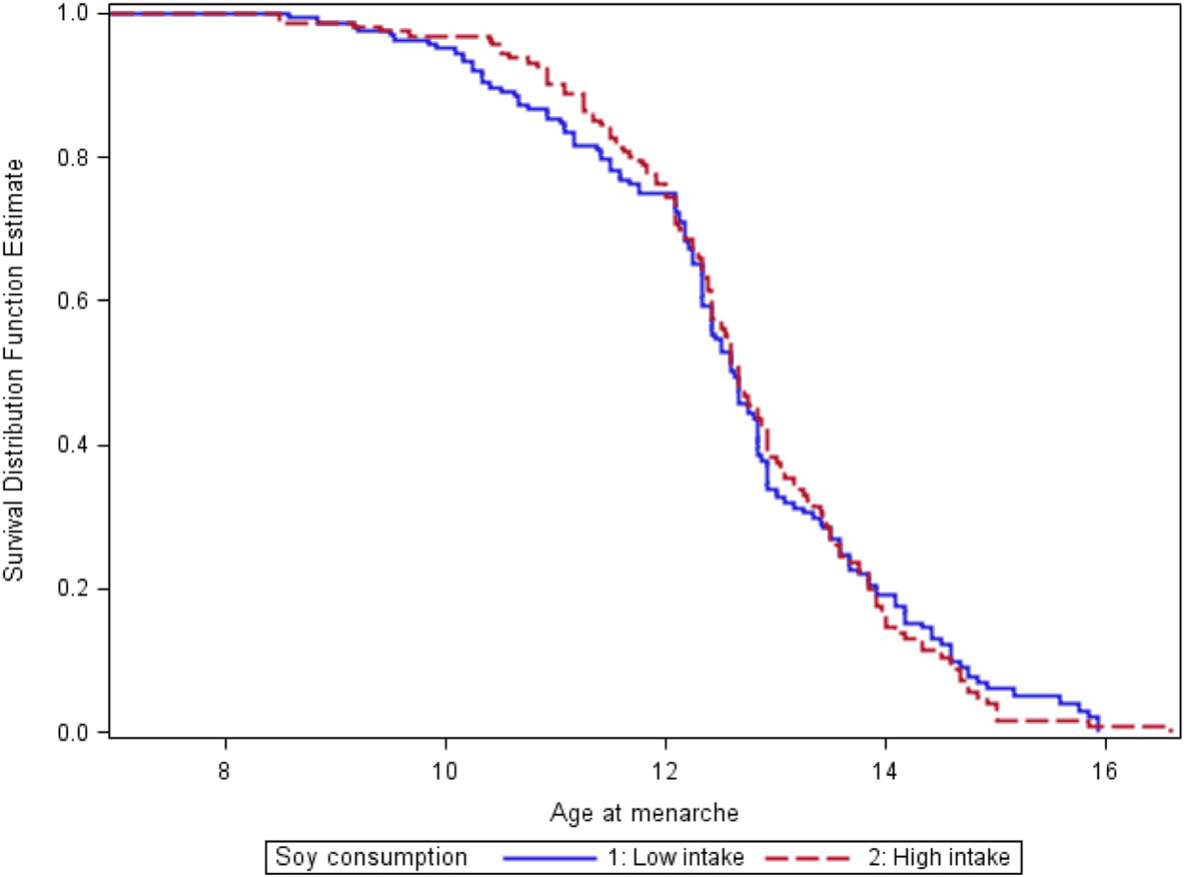
**Age at menarche survival curves for low- and high-soy consumers.** Legend: Kaplan-Meier survival curves are shown for both low and high consumers of soy. Median AOMs are 12.67 (low intake) vs. 12.58 (high intake) for B (p_log rank test_ = 0.84). Values are energy-adjusted. Age at menarche is in years.

Results of the Cox proportional hazards regression for the occurrence of menarche according to consumption of total soy and the 3 types of soy foods at both the continuous and categorical levels are presented in Table 3. The risk of occurrence of menarche was not significantly related to soy food consumption. Moreover, risk did not significantly differ according to level of consumption for the 3 types of soy foods and the combined intake (total soy) when adjusted for total food intake, age, study site, type of school, mother’s and father’s educational levels, mother’s and father’s ethnicities, and meat intake. Further adjustment for BMIz did not result in substantial changes in point estimates nor significance levels; thus, only results for the model which includes BMIz was shown (Table 3). The Kaplan-Meier curves (Figure 1) which plot the AOM for two energy-adjusted levels of total soy consumption show that low-consumers (median AOM=12.67 y) and high-consumers (median AOM=12.58 y) have no significant median AOM differences.

**Table 3:** **Relative risks**^**a **^**for the occurrence of menarche according to total soy and soy foods consumption at the continuous and categorical levels**

	**Continuous level (1/day)**	**p-value**	**Level of soy consumption**^**b**^			
			**Quartile 1**	**Quartile 2**	**Quartile 3**	**Quartile 4**
**Total soy**						
Median intake, svg/wk			0.0	4.5	13.5	24.2
Median AOM, yr^c^			12.6	12.4	12.5	12.6
Relative risk	0.99 (0.91, 1.07)	0.770	1.00 Ref	0.96 (0.66, 1.40)	0.95 (0.64, 1.41)	1.19 (0.79, 1.80)
**Meat alternatives**						
Median intake, svg/wk			0.0	2.5	8.0	14.5
Median AOM, yr^c^			12.5	12.5 (12.5)	12.8 (12.7)	12.5
Relative risk	1.01 (0.90, 1.13)	0.838	1.00 Ref	0.92 (0.62, 1.36)	1.02 (0.70, 1.48)	1.07 (0.70, 1.65)
			**Non-consumers**	**Low-moderate**		**High**
**Tofu/Traditional soy**						
Median intake, svg/wk			0.0	1.0		4.8
Median AOM, yr^c^			12.5	12.6		12.4
Relative Risk	0.90 (0.70, 1.16)	0.401	1.00 Ref	0.93 (0.68, 1.26)		1.00 (0.72, 1.40)
**Soy beverages**						
Median intake, svg/wk			0.0	1.5		8.5
Median AOM, yr^c^			12.6	12.4		12.6
Relative Risk	0.98 (0.84, 1.14)	0.759	1.00 Ref	1.07 (0.79, 1.46)		1.12 (0.82, 1.54)

^a^Cox proportional hazards regression model adjusted for total food intake, age, site, type of school, mother’s and father’s education, mother’s and father’s ethnicity, meat intake, and BMI z-scores.

^b^Levels of consumption for total soy and meat alternatives are in quartiles while that for tofu/traditional soy and soy beverages are categorized as non-consumers (“0” soy consumption), low-to-moderate consumers (≤75th percentile excluding non-consumers) and high consumers (>75th percentile) due to more skewed distributions.

^c^AOM = age at onset of menarche, excludes those who have not reached menarche.

To further determine if soy consumption groups differed in the timing of their puberty, we performed modified Poison regression. Results show that in all categories of soy foods, there were no significant linear trends in risk for either early AOM or late AOM as intake level proportionally increased (Table 4). However, when the three types of soy foods were combined (total soy), a significant early AOM risk was found among those in the second quartile (Q2) of total soy consumption (RR: 1.85 [95% CI: 1.01, 3.40], p = 0.047) compared to the reference group, Quartile 1. No significant changes in risk for late AOM were observable even if there seems to be a decreasing risk for late menarche as soy beverage intake increases.

**Table 4:** **Relative risks (95% confidence interval)**^**a **^**for early onset (early AOM) or late onset (late AOM)**^**b **^**of menarche according to levels of total soy and soy foods consumption**

	**Level of Consumption**^**c**^			
	**Quartile 1**	**Quartile 2**	**Quartile 3**	**Quartile 4**
**Total soy intake**				
Early AOM	1.00 Ref	1.85 (1.01, 3.40)^d^	1.62 (0.80, 3.26)	1.85 (0.91, 3.75)
Late AOM	1.00 Ref	1.17 (0.53, 2.60)	0.86 (0.32, 2.30)	0.83 (0.30, 2.30)
**Meat alternatives**				
Early AOM	1.00 Ref	1.45 (0.79, 2.66)	1.08 (0.53, 2.19)	1.54 (0.77, 3.08)
Late AOM	1.00 Ref	1.22 (0.56, 2.65)	0.81 (0.32, 2.06)	0.96 (0.36, 2.57)
	**Non-consumers**		**Low-moderate**	**High**
**Traditional soy**				
Early AOM	1.00 Ref		1.30 (0.81, 2.08)	1.39 (0.81, 2.38)
Late AOM	1.00 Ref		1.30 (0.70, 2.41)	1.05 (0.50, 2.23)
**Soy beverages**				
Early AOM	1.00 Ref		1.41 (0.88, 2.28)	1.15 (0.66, 2.02)
Late AOM	1.00 Ref		0.95 (0.52, 1.74)	0.70 (0.31, 1.57)

^a^Modified Poisson regression model adjusted for total food intake, age, site, type of school, mother’s and father’s education, mother’s and father’s ethnicity, meat intake, and BMI.

^b^Early AOM defined as AOM < 12.0 years and late AOM as AOM ≥ 14.0 years.

^c^Levels of consumption for total soy and meat alternatives are in quartiles while that for tofu/traditional soy and soy beverages are categorized as *non-consumers* (“0” soy consumption), *low-to-moderate consumers* (≤75^th^ percentile excluding *non-consumers*) and *high consumers* (>75^th^ percentile) due to more skewed distributions.

^d^p = .047.

Certain covariates were found to be associated with AOM (not shown). BMI was consistently related to menarche (RR: 1.20 [95% CI: 1.06, 1.35], p = 0.004) and girls whose mother have a college education or higher seem to be protected from late AOM (RR: 0.37 [95% CI: 0.16, 0.84], p = 0.019).

## Discussion

We did not find significant associations between soy intake and AOM in our study population of adolescent Adventist girls who have a wide range of soy intake. The risk for an early or late AOM was not significantly different for the high versus the low soy consumers. The lack of significant associations was consistent across the different soy food types and for the combined total intake of soy foods. The biggest crude difference in the AOM between any soy consumption level groups in our study was 0.2 year (~2.4 months), and 0.1 year (~1.2 months) when adjusted for relevant covariates. A previous study reported a non-significant 4-month difference in AOM between those who were fed and those who were not fed soy formula during early infancy [22]. This larger difference was not significant, probably because of the small number of girls exposed to soy formula. On the other hand, our study involved a larger number of girls exposed and not exposed to soy foods; nevertheless, the AOM difference found in our study was smaller.

As expected, our study population has a wide range of soy consumption, from 0 intake to several servings per day (mean [SD] = 12.9 [14.4] svg/wk), with meat alternatives comprising more than half of the soy foods consumed. While there is a wide range in intake of tofu/traditional soy foods and soy beverages, these are less commonly consumed by this group compared to meat alternatives. About 20% drink soy beverages ≥1×/day while ~23% eat tofu and traditional soy foods ≥3×/week whereas ~54% eat meat alternatives ≥4×/wk.

We made the assumption that for our study population, it was better to assess current soy intake than to retrospectively assess soy intake before onset of menarche. Compared to adults, dietary assessment among children and adolescents is more prone to errors [34] and these errors can be compounded when recalling past consumption. Although it is presumed that dietary intake may change due to increased independence as children transition to adolescence, there is evidence to the contrary. In a study done on a diverse population of children ages 9–18 years, dietary intake patterns that were assessed 5 years apart have been found to be stable over time [35]. For decades, soy foods and beverages have been readily available in Adventist school cafeterias and local groceries near communities with an Adventist presence. Consumption of these foods among Adventists is not a recent fad but a lifestyle some families choose, or choose not to follow. In our web-based questionnaire, one section asked participants to report their intake of selected food items—which include tofu, meat alternatives, soymilk, and soy ice cream—when they were 8–10 years old relative to their current intake. Participants reported whether they *never/rarely* ate or ate these foods *less than, the same,* or *more than* their current intake. We found that current intake estimates were 61- 80% in agreement with past consumption. This confirms the relative stability of soy foods intake in this population and *a posteriori* justifies our decision to ask participants to report their current intake. We believe this also relieved the undue burden of recalling childhood dietary intake and diminished measurement errors.

This report focused on exposure to soy as a food in its various forms. Previous soy-related studies have focused on one of its components, isoflavones. Aside from isoflavones, soy foods are also rich sources of high-quality plant proteins, omega-6 and omega-3 polyunsaturated fats, dietary fiber, and other phytochemicals. Some of these nutrients had been implicated in pubertal development: Higher vegetable protein intake during childhood had been found to be related to later onset of menarche [27,36]; dietary fiber was found to be an important factor in the hormonal maturation of pubertal girls [28] and associated with a delay in menarcheal age [29]; and, high dietary fat intake was found to be associated with early menarche [30]. Thus, several soy food compounds may synergistically impact the onset of menarche; that is, the effect of the food can be different from the effect of the sum of its components, and certainly, different from any single component. Consequently, a food approach has scientific merit, and may have direct practical applications for the consumer as well as the guided professional advice.

To our knowledge, this is the first study on the intake of soy foods during adolescence and AOM. Two previous studies looked at exposure to soy formula during infancy. Adgent et al. in the Avon Longitudinal Study of Parents and Children reported an earlier risk of menarche among girls who were fed soy formula in infancy; however, the 4-month median difference in AOM between those fed soy formula (149 mos or 12.4 yr) and those fed non-soy formula (153 mos or 12.8 yr) was not significant [22]. A retrospective cohort study that also looked at soy formula exposure during infancy did not find any difference between the AOM of soy-formula fed (mean = 12.6 years) and of cow milk formula-fed (mean = 12.7 years) participants [24]. The Dortmund Nutritional and Anthropometric Longitudinally Designed study assessed isoflavone exposure during childhood (before pubertal growth spurt). In a sub-sample of 119 white girls, no significant 4-month AOM difference was found between the highest and the lowest tertiles of isoflavone exposure [23].

A panel that examined US national data from 1940 to 1994 on puberty timing concluded that AOM has been decreasing [37]. At the same time, the use of soy-based formula and soy foods had increased in the US over the last few decades. Are these secular trends related? The mean AOM (12.5 years) of our high soy-consuming study population (born between 1994 and 2001) is the same as or slightly higher than the mean AOM of US girls (12.4 years) born 15 years earlier (1980–85) [1], which argues against a connection between these trends.

Evidence suggests that higher pre-pubertal BMI may influence the rate of pubertal development and earlier attainment of menarche [13]. BMI may be an intermediate variable in the causal path between diet and maturation [14,38] but in our study, we did not have pre-pubertal BMI information so we used measured (current) BMI. We found BMI to be significantly associated with early menarche. We cannot discount the possibility that increase in body size—and the corresponding increase in BMI—associated with pubertal changes is more prominent among those who had earlier menarche in our study, which may explain this association between BMI and early AOM.

One of the strengths of our study is that our female adolescent population has a wide range of soy intake, the majority of whom come from families that have relatively stable dietary patterns over the years [39,40]. The wide range of soy intake increases the power to find significant relationships, if they exist. Another strength is the use of an age-appropriate instrument for data collection with a web-based FFQ that assessed soy consumption with acceptable validity. Our study also has several weaknesses that should be considered when interpreting our findings. Although the extensive soy exposure may contribute to the ability to see significant differences between consumers and non-consumers, the sample size may not have been enough to see a significant 2-month difference in AOM. The cross-sectional design of the study precludes a firm temporal connection between soy intake and AOM. A prospective assessment of soy consumption in childhood may have provided a better dietary measurement than the current consumption that we used in our study. Additionally, the use of a web-based food frequency questionnaire is subject to the same inaccuracies in self-reporting intake as paper-based questionnaires, and thus, the purported error may have contributed to the null findings. The use of current BMI instead of pre-pubertal BMI is also a limitation due to the aforementioned reasons.

## Conclusions

Our study findings indicate that soy consumption around the time of puberty is not associated with the AOM. The mean AOM of this population—which has a wide range of and relatively higher soy intake than the general U.S. population—is similar or slightly higher than the national norm. This suggests that the increased popularity of soy foods may not be related to the decreasing secular trend in menarcheal age among U.S. girls.

## Competing interests

MM regularly consults for companies that manufacture and/or sell soy foods. The other authors have no conflict of interest to declare.

## Authors’ contributions

JS conceived, designed and directed the study, and critically reviewed the manuscript; KO analyzed the data; MM critically reviewed and edited the manuscript; PP supervised the data collection and management for the Michigan side, and GSS designed the study with JS, supervised the data collection and management, and wrote the manuscript. All authors contributed to the editing and approval of the submitted manuscript.

## Supplementary Material

Additional file 1 Food frequency questionnaire items. Click here for file
