# Supplementary material for: Is soy intake related to age at onset of menarche? A cross-sectional study among adolescents with a wide range of soy food consumption
Source: Nutr J. 2014 Jun 3;13:54. doi: 10.1186/1475-2891-13-54 (PMC4051381; doi:10.1186/1475-2891-13-54)
Supplement: Additional file 1 — Food frequency questionnaire items. [file 1475-2891-13-54-S1.docx]

**Additional file 1.** Food frequency questionnaire items

| **FOOD GROUP** | **FOOD ITEMS** |
| --- | --- |
| **Convenience foods** | meat burger, *vegeburger*, other meatless burgers, real meat hotdog, *vegemeat hotdog*, real meat sandwich, *vegemeat sandwich*, peanut butter sandwich, egg salad sandwich, cheese sandwich, real meat breakfast sandwich, *vegetarian breakfast sandwich*, egg and cheese breakfast sandwich, real meat burrito, meatless burrito, real meat fish/taco, *vegemeat taco*, bean and cheese taco, cheese only quesadilla, *quesadilla with vegemeat*, quesadilla with real meat, cheese only pizza, vegetables pizza, real meat pizza, vegetables only egg/spring rolls, *egg/spring rolls with tofu*, egg/spring rolls with real meat, vegetarian sushi, *imitation fish/seafood sushi*, real fish/seafood sushi, meatless nachos, nachos with real meat |
| **Protein Foods** | vegetable dish with real meat, *vegetable dish with vegemeat*, *vegetable dish with tofu*, pasta/noodles with real meat, *pasta/noodles with vegemeat, pasta/noodles with tofu*, rice dish with real meat, *rice dish with vegemeat, rice dish with tofu*, bean dish with real meat, *bean dish with vegemeat, bean dish with tofu, tofu, tofu soups, tempeh*, real meat patty, *vegemeat patty*, other vegetarian patty, real chicken, *vegechicken*, real meat steak/roast, *vegemeat steak/roast*, real meat meatloaf/meatball, *vegemeat loaf/meatball*, real meat link/frank, *vegelink/vegefrank*, real eggs, real fish, *vegetarian fish* |
| **Beverages** | Water, sports drinks, regular soda, diet soda, regular milk, low-fat milk, non-fat milk, *plain soymilk, flavored soymilk, homemade soymilk*, almond milk, rice milk, 100% real fruit juice, fruit drinks, smoothie/shake with milk, *smoothie/shake with soymilk/soy protein powder*, smoothie/shake with whey protein powder, smoothie/shake with fruit juice only, black coffee, coffee blended with dairy, *coffee blended with soymilk*, sugar-sweetened tea, artificially sweetened tea, unsweetened tea |
| **Starches/**  **Cereals** | regular bread, flat bread, bagel/English muffins, fried potatoes/French fries, baked/boiled/canned potatoes, mashed potatoes, pasta/noodles with red sauce, pasta/noodles with white sauce, mac and cheese, ramen noodles |
| **Vegetables/**  **Fruits** | Melons, apples/pears, peaches/plums/nectarines, oranges, grapes, berries, tropical fruits, banana, dried fruits, canned/stewed fruits and sauces, avocado and guacamole, leafy green vegetables, salads and salad greens, snack vegetables, cabbage family, corn and peas, green and string beans, squash, eggplant, peppers, tomatoes |
| **Snacks/ Sweets** | Popcorn, potato chips, corn/tortilla chips, other chips, nuts and nut butters, other snacks, *soy nuts*, pastries, donuts, cakes/cookies, candies |
| **Soups/**  **Legumes** | Mixed bean soups, lentils/split pea soups, haystack, hummus, *edamame*, cream-based soups, broth-based soups |
| **Dairy Products** | Dairy-based ice cream, *soy-based ice cream*, dairy-based yogurt, *soy-based yogurt*, dairy-based cheese, *soy-based cheese*, dairy-based creams/spreadable and cottage cheese, *soy-based creams/spreadable and cottage cheese*, butter, margarine |
